# Data driven multiscale modelling of paroxysmal brain transitions using DC-coupled electrophysiological data

**DOI:** 10.1371/journal.pone.0353399

**Published:** 2026-07-17

**Authors:** Amirhossein Jafarian, Rob C. Wykes

**Affiliations:** 1 MRC Cognition and Brain Sciences Unit, University of Cambridge, Cambridge, United Kingdom; 2 Research Department of Epilepsy, UCL Queen Square Institute of Neurology, London, United Kingdom; 3 Centre for Nanotechnology in Medicine & Division of Neuroscience, University of Manchester, Manchester, United Kingdom; Belgrade University Faculty of Medicine, SERBIA

## Abstract

We introduce a novel parameter estimation framework for a slow-fast neuronal model using DC-coupled electrophysiological data recorded from the WAG-Rij rat model of generalised seizures. In this animal model, fluctuations in extracellular potassium concentrations are hypothesised to drive infra-slow oscillations (ISO) that precede spike-wave discharges. We construct a biophysically motivated slow-fast dynamical system in which seizures are triggered by fluctuations in extracellular potassium concentrations to model the in vivo observations. Specifically, we interpret ISOs dynamics (mathematically) as the integral transform (or low-pass filter) of extracellular potassium concentrations, facilitating real time tracking of physiological states. Model parameters are estimated from empirical data, using an expectation-maximisation approach that optimises a regularised likelihood function, while biological states are inferred through the unscented Kalman filter. The inferred model allows tracking changes in latent proxy of extracellular potassium concentrations from DC-coupled electrophysiological recordings (exhibiting paroxysmal transitions) under the assumption that in our preclinical model extracellular potassium dynamics contribute to seizure generation. We validate the consistency of inferred hidden biological states across longer datasets containing multiple seizure events that were not utilised during parameter estimation. The results demonstrate that ISOs provide sufficient information to infer latent ionic dynamics and support the conceptualisation of seizure onset as bifurcation-driven transitions modulated by the ionic changes.

## Introduction

Infra-slow neuronal oscillations (ISOs), defined as brain activity in the frequency range [0.01, 0.1] Hz [1] encode critical information about brain dynamics including sleep regulation [2–5], resting-state networks [6,7], and neurological conditions such as epilepsy [8–11]. These slow oscillations are thought to arise from after-hyperpolarisation of ionic concentrations [12–17]; long-lasting hyperpolarising potentials associated with astrocyte buffering of potassium dynamics [18–21]); and network dynamics [22–24]. Unlike conventional AC-coupled recordings, which capture high-pass filtered neuronal signals, DC-coupled recordings detect neuronal activity across a wider frequency range, including ISO, offering a valuable biomarker for seizure transition [10]. We recently demonstrated that the use of DC-coupled electrophysiological recordings substantially enhances the precision and translational relevance of neuronal models of seizure dynamics [25].

In this study, we provide a data-driven framework for modelling laminar DC-coupled recordings from the WAG-Rij rat model of generalised seizures [10]. These recordings exhibit quasi-periodic paroxysmal transitions, that coincide with the rising phase of ISOs. We hypothesise that ISOs are mathematically proportional to the leaky integral transform (or low pass filter) of extracellular potassium concentrations, which modulate seizure susceptibility. Our approach therefore should be considered as hypothesis-driven identification framework, rather than as direct measurements of extracellular potassium concentrations.

To capture the phenomenology of paroxysmal transitions in the animal model, we combine a well-established cortical column model [26,27] with laminar-specific slow extracellular potassium concentrations (${\left[K^{+}\right]}_{ex})$ changes [28,29] building on prior empirical evidence, e.g., [30–32]. The model separates fast neuronal dynamics (e.g., membrane potentials and synaptic activity) from activity-dependent potassium concentration dynamics enabling paroxysmal transitions autonomously, e.g., [29,33–40]. The slow ${\left[K^{+}\right]}_{ex}\;$dynamics guide the fast subsystem across bifurcation points, leading to limit cycles or return to resting states. The bifurcation structure of the slow-fast model allows for smooth or abrupt transitions depending on ${\left[K^{+}\right]}_{ex}$, reflecting the heterogeneous seizure onset patterns observed in vivo.

Dysregulation of extracellular potassium concentrations could result in active DC shifts and ISO’s in electrophysiological recordings, which may contribute to seizure initiation [18,28,30–32]. During the pre-ictal phase, neurons begin to fire rapidly but remain desynchronised, making their effects on local field potentials (LFPs) subtle. However, as neuronal firing rates increase, extracellular potassium concentrations are expected to increase, potentially contributing to the DC shift in LFP just before seizure onset [41]. Once the extracellular potassium concentration approaches or crosses a critical threshold, seizures are triggered. Alternatively impaired potassium buffering or clearance following neuronal activity elevates extracellular potassium constrain, and its slow removal by astrocytes and pumps causes rhythmic build-up and clearance. This cycle of excitability and recovery generates ISO’s in the LFP. Our core hypothesis is that ISOs serve as an observable proxy for the underlying potassium concentrations dynamics in this model of absence epilepsy. By using DC-coupled data and modelling ISOs as proxy of changes in extracellular potassium we develop a novel hypothesis-driven inference scheme for the multiscale slow-fast model of paroxysmal transitions.

**Fig 1:**
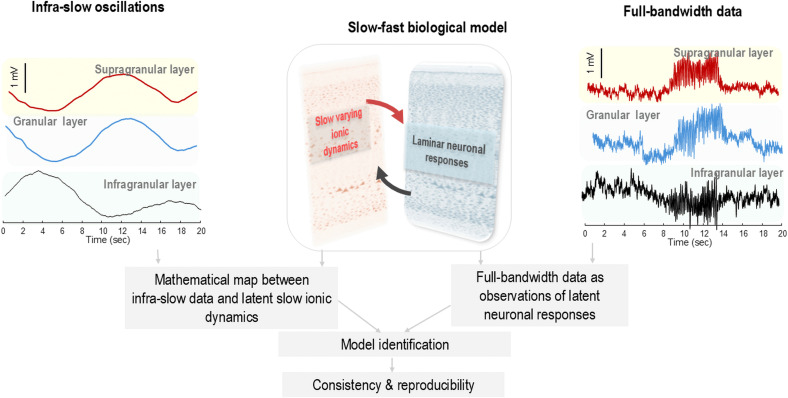
Schematic overview of the modelling and inference framework. The central panel illustrates the proposed slow-fast model used to capture paroxysmal transitions in the WAG-Rij rat animal model of absence epilepsy. The neuronal dynamics within each cortical layer are regulated by layer-specific extracellular potassium concentrations. These ionic concentrations evolve on a slower timescale and act as modulators of neuronal excitability, enabling transitions into and out of seizure states. The right panel shows empirical DC-coupled full-bandwidth recordings treated as the readouts of fast neuronal states (e.g., synaptic and membrane dynamics). The left panel shows infra-slow oscillations (i.e., 0.01–0.1 Hz), mathematically modelled as the low passed transforms of laminar potassium concentrations (i.e., a proxy measurement rather than the concentration itself). A nonlinear state-space model identification is applied to infer latent variables of the model. Finally, we assess the consistency and reproducibility of the inferred dynamics using held-out data segments, not used during the model tuning.

A graphical illustration of the data-driven modelling framework is shown in Fig 1, comprising three main components: (i) specification of auxiliary observation equations (‘slow observations’) that mathematically link slow states to infra-slow oscillations; (ii) application of a regularised likelihood approach for data-driven modelling; and (iii) demonstration of the consistency and reproducibility of latent state estimation on unseen data. Glossaries of model variables are provided through Table 1–3.

**Table 1:** Acronyms.

Acronyms	Description
EM	Expectation-maximisation.
AIC	Akaike information criterion.
FB(mV)	Full-bandwidth data.
LFP(mV)	Local field potential.
UKF	Unscented Kalman filter.
ISO(mV)	Infra-slow oscillations.
DC (mV)	Direct current.
AC (mV)	Alternating current.
NMDA	N-methyl-D-aspartate receptors.
AMPA	α-amino-3-hydroxy-5-methyl-4-isoxazolepropionic acid.
GABA	Gamma-Aminobutyric Acid.
[$Mg^{+}](Mm)$	Magnesium concentration.
$\left[{Na}^{+}\right]$(Mm)	Sodium concentration.
${\left[K^{+}\right]}_{ex}$(Mm)	Extracellular potassium concentration.
sg, g, andig	Supragranular, granular and infra granular layers of cortical column.
L,l	Sensor gains and scale factor between slow-states and ISO.
SP, SS, II, DP	Superficial pyramidal cells, spiny stellate excitatory neurons, interneurons, deep pyramidal cells, respectively.
SWD	spike-wave discharge
ATPase	Adenosine 5’-TriPhosphatase, adenylpyrophosphatase

**Table 2:** Glossary of time dependent variables in the slow-fast conductance-based model.

Variable (unit)	Description
n(t)~N(μ, Σ) (Hz)	Exogenous inputs, modelled as random signals with normal distributions where μ is the mean firing rates and Σ is the covariance of the noise.
V(mV)	Mean depolarisation of a neuronal population.
S(.)(Hz)	Firing rates as the sigmoid function of depolarisation with threshold −40 mV.

**Table 3:** Parameters of the neuronal model (see also Fig 2).

Variable (unit)	Description	Parameterisation/value	Mean	Standard deviation
κ (Hz)	Rate constants of AMPA, GABA, and NMDA.	κ=[4,16,100]	Fixed	
$C\;(\frac{\mu F}{cm^{\text{2}}})$	Membrane capacitance of SS, SP, II, and DP neurons.	C=[128 128 256 32]/1000	Fixed	
$H_{i}$	Inhibitory connections.	$H_{i}=[\begin{array}{cccc} 0 & 0 & 2 & 0\\ 0 & 0 & 2 & 0\\ 0 & 0 & 0 & 0\\ 0 & 0 & 8 & 0 \end{array}]$	Fixed	
$H_{nmda/ampa}$	Excitatory NMDA/AMPA connections.	$\;H_{nmda/ampa}=[\begin{array}{cccc} 0 & 0 & 0 & 0\\ 4 & 0 & 0 & 0\\ 4 & 0 & 0 & 2\\ 0 & 4 & 0 & 0 \end{array}]$	Fixed	
$n_{V}(t)(Hz)$	Input to excitatory cells $\;n_{V}(t)\sim \;N(\mu_{V},\;\Sigma_{V})$.	$\text{3}\;\times exp(\mu_{V})$ $\text{0}.\text{1}\;\times exp(\Sigma_{V})$	$n_{V}(t)\sim \;N(\text{2}.\text{6},\;\text{0}.\text{5})$	±(3.3, .07)
${n_{\left[K^{+}\right]}}_{ex}(t)$	Random fluctuations in ${\left[K^{+}\right]}_{ex}\;$ modelled as $n_{{\left[k^{+}\right]}_{ex}}(t)\sim \;N(\text{0},\;\Sigma_{{\left[K^{+}\right]}_{ex}})$.	$\text{0}.\text{0}\text{1}\;\times exp({\Sigma_{\left[K^{+}\right]}}_{ex})$	$n_{t}\sim \;N(\text{0},\;\text{0}.\text{0}\text{2})$	±(0.12)
J(mV)	Neural contributions of distal layers to local neurons.	$J_{c}\times exp(J)$ $J_{c}=[\begin{array}{cc} .\text{5} & .\text{2}\\ .\text{5} & .\text{5}\\ .\text{2} & .\text{5} \end{array}]\%$	$J=[\begin{array}{cc} \text{0}.\text{2}\text{7} & \text{0}.\text{1}\\ \text{0}.\text{3}\text{7} & \text{0}.\text{4}\text{1}\;\\ \text{0}.\text{2}\text{1} & \text{0}.\text{5}\text{5} \end{array}]\%$	$\pm [\begin{array}{cc} 0.36 & 0.25\\ 0.41 & 0.85\\ \text{0}.\text{1}\text{1} & \text{0}.\text{4}\text{9} \end{array}]$
β	Layer specific slow to fast connections.	0.5 ×exp(β)	$\beta =[\begin{array}{c} 0.12\\ 0.11\\ 0.33 \end{array}]$	$\pm [\begin{array}{c} \text{0}.\text{1}\text{3}\\ \text{0}.\text{1}\text{7}\\ \text{0}.\text{4}\text{2} \end{array}]$
γ(Hz)	Layer specific time constant of potassium concentrations.	0.01 ×exp(γ)	$\;\gamma =[\begin{array}{c} \;\text{0}.\text{0}\text{5}\\ \text{0}.\text{0}\text{6}\\ \text{0}.\text{0}\text{1} \end{array}]$	$\pm [\begin{array}{c} \text{0}.\text{2}\\ \text{0}.\text{2}\\ \text{0}.\text{1} \end{array}]$
$e_{iso}$	ISO to slow states random effect: $e_{iso}\sim \;N(\text{0},\;\Sigma_{*iso})$.	$\Sigma_{iso}\times exp(\Sigma_{i})$ $\Sigma_{iso}=[\begin{array}{c} \text{2}\\ \text{2}\\ \text{2} \end{array}]$	$\Sigma_{i}=[\begin{array}{c} \text{0}.\text{0}\text{3}\\ \text{0}.\text{0}\text{5}\\ \text{0}.\text{0}\text{2} \end{array}]$	$\pm [\begin{array}{c} \text{0}.\text{1}\text{9}\\ \text{0}.\text{7}\text{4}\\ \text{0}.\text{5}\text{1} \end{array}]$
$e_{FB}$	Layer specific sensor noise: $e_{FB}\sim \;N(\text{0},\;\Sigma_{*FB})\;$.	${\Sigma_{FB}\times exp(\Sigma }_{F})$ $\Sigma_{FB}=[\begin{array}{c} \;\text{2}\;\\ \;\text{2}\;\\ \;\text{9}\; \end{array}]$	$\Sigma_{F}=[\begin{array}{c} \text{0}.\text{3}\\ \text{1}.\text{3}\\ \text{3}.\text{1} \end{array}]$	$\pm [\begin{array}{c} \text{0}.\text{2}\text{8}\\ \text{0}.\text{6}\text{6}\\ \text{0}.\text{3}\text{4} \end{array}]$
ϵ	Time scale separation between slow and fast states.	0.17 ×exp(ϵ)	ϵ= 0.25	± 0.2
$\left[V_{L_{c}},\;V_{K},\;V_{NMDA}\;{,V}_{AMPA},\;V_{GABA}](mV\right)$	Reversal equilibrium potentials.	[−70, −96, 10, 60, −90]	Fixed	

The (i,j) element in the matrices associated with parametrisation of intrinsic connections Σ, means connections that originate from a population j and target population i in a region (row and column in the matrix elements span from 1 to 4 and associate with excitatory/inhibitory connections amongst SS, SP, II and DP layers, respectively). Unknown parameters have default values multiplied by the exponential of their estimated values from empirical data. Parameters that are fixed have been written in the usual format.

## Materials and methods

### Animal model of seizures

We studied DC-coupled electrophysiological data obtained from the WAG-Rij rat model of absence epilepsy, characterised by generalised spike and wave seizures discharges (SWDs) [10]. In brief, WAG-Rij rats were implanted with a 16-channel graphene micro-transistor array for recordings through cortical layers of the somatosensory cortex. Recording sessions in awake rats lasted 10–60 minutes and occurred twice weekly for ten weeks. All experiments were approved by the Home Office (license PPL70–13691) and the University College London Queen Square Institute of Neurology, Animal Welfare Ethical Review Body within the University College London.

Graphene solution-gated field-effect transistors exhibit excellent DC stability and low in vivo drift, enabling high-fidelity recording of DC shifts and infra-slow activity compared with conventional metal microelectrodes [42]. Our recordings showed stable baselines over the duration of the sessions, allowing reliable separation of structured infra-slow oscillatory events from any residual non-biological drift. Infra-slow activity was extracted from the DC-coupled data using a band-limited baseline-removal procedure where ultra-slow baseline drift was removed using a 0.001 Hz high-pass/baseline-removal filter, and faster electrophysiological activity was removed using a 0.1 Hz low-pass filter. This yielded the empirical ISO signal.

In adult WAG-Rij rats, cortical electrophysiological recordings display SWDs oscillating between ~7 −10 Hz, with episodes from a few seconds up to 40 seconds. By six months of age, these rats typically experience hundreds of SWDs daily. WAG-Rij rats also exhibit subtle behavioural changes during SWDs including facial muscle twitches, rapid breathing, head movements, and eye fluttering.

### Multiscale model of seizures

To model electrophysiological data that undergo seizure transitions, we developed a phenomenological laminar-specific, conductance-based cortical column model that incorporates slow ionic-concentrations dynamics. This model extends prior works [26,27] by including activity-dependent extracellular potassium regulation, e.g., [29,40,43] following evidence that changes in ${\left[K^{+}\right]}_{ex}$ modulate neuronal excitability. We express the model in a slow–fast state-space form as follows:


$$\begin{array}{c} \left\{ \begin{array}{c} @l\frac{dx}{dt\;}=f(x,\theta )+n_{x}(t)\;\\ \frac{d\theta }{dt}\;=\epsilon g(x,\theta )+n_{\theta }(t) \end{array}\right. \end{array}$$ (1)

$$\begin{array}{c} FB=\text{ L}(x)+e_{FB} \end{array}$$ (1-2)

$$\begin{array}{c} ISO=\text{l}(F\int \theta \;dt)+e_{ISO} \end{array}$$ (1-3)

In Equation (1), x is fast time scale biological processes (e.g., membrane potentials, conductance dynamics, firing rates and synaptic responses) and θ denotes the slow states (extracellular potassium concentrations dynamics) and 0<ϵ≪1 sets the time-scale separation. Distal presynaptic inputs to fast-states are modelled as random fluctuations with Gaussian distributions, with mean firing rates $\mu_{x}$ and (small) noise covariance $\Sigma_{x}$, $n_{x}(t)\sim N(\mu_{x},\;\Sigma_{x})$, while slow-states are driven by (small) random perturbations $n_{\theta }(t)\sim N(\text{0},\;\Sigma_{\theta })$ (i.e., covariance of noise $\Sigma_{\theta }$ is small) which resembling small changes in the synaptic environments [44,45].

Equation (1-2) relates full-bandwidth recordings (denoted by FB) and the hidden biological states x through scaling factors L. Equation (1-3) explains the relation between infra-slow oscillations (ISO) and the transformation of extracellular potassium concentrations, scaled by constant l. The operator F∫(.)dt implements leaky integrator with characteristic cutoff frequency of 0.01 Hz. The observer equation in turn reflecting build up and clearance of ionic dynamics rather than a monotonic accumulation. In practice, to implement leaky integrator, one can augment the generative model with an auxiliary state z defined as the leaky transform of the latent slow state. The evolution of z is governed by $\frac{dz}{dt}=\frac{\text{1}}{\tau }(-z+\theta )$ with $f_{c}=\frac{\text{1}}{\text{2}\pi \tau }=\text{0}.\text{0}\text{1}$ Hz.

Note that, ISO is an indirect signature (a filtered proxy) of a latent ionic state, not the concentration itself. However, such observer equation facilitates data driven modelling.

The response of a neuronal model to the external inputs can be understood as a low pass filtering operation [46–48]. The time scale separation ensures, slow states change negligibly to that of neuronal dynamics over a short time window—i.e., on a finite interval one can assume on average, slow dynamics are approximately constants [49]. This implies, a low pass filtered version of the slow states is observable in the output of the neuronal model.

The model of sensor noise and/or model inadequacy are denoted by $e_{FB}$, $e_{ISO}$ in equation (1-2) and (1-3), which are given by independent identically distributed Gaussian distributions with zero mean and unknown variances, i.e., $e_{FB}\sim N(\text{0},\;\Sigma_{FB})$ and $e_{ISO}\sim N(\text{0},\;\Sigma_{ISO})$.

**Fig 2:**
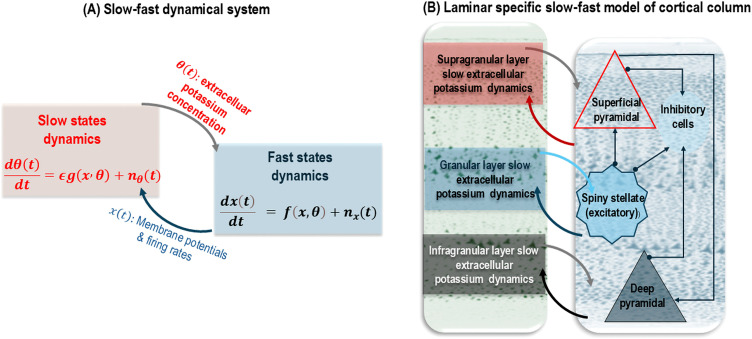
Layer-specific slow-fast model of cortical dynamics. Left panel: schematic of the proposed slow-fast dynamical system. Fast biological states (synaptic activity or membrane potentials) x and slow ionic concentration θ (e.g., potassium concentration) interact through bidirectional feedback, forming a regulatory mechanism mediated by time scale separation constant 0<ε≪1. Disruptions of this regulation is hypothesised to underlie paroxysmal transitions. Right panel: cortical-column circuit with four neuronal populations: superficial pyramidal cells (SP), spiny stellate excitatory cells (SS), deep pyramidal neurons (DP) and inhibitory interneurons spanning supragranular, granular, and infragranular layers, with connectivity parameters detailed in Table 3. Each population is modelled with Morris–Lecar neuronal model equipped with slow extracellular potassium concentration dynamics. Layer-specific interactions between slow and fast states enable the generation of ISO and transitions into and out of seizures across the layers by the model.


The neuronal dynamics associated with each cortical layer in Fig 2 is governed by the conductance-based Morris–Lecar models with a slow ${\left[K^{+}\right]}_{ex}$ as follows:

$$\begin{array}{cc} \;\frac{dV}{dt}=\; & {\frac{\text{1}}{C}[g}_{L_{c}}\left(V_{L_{c}}-V\right)+S(V)\;{\left[K^{+}\right]}_{ex}\left(\;V_{K}-V\right)+\;g_{AMPA}\left(V_{AMPA}-V\right)+g_{GABA}\left(V_{GABA}-V\right)\\ & \;+g_{NMDA}\;S_{\left[Mg^{+}\right]}\;(V)\left(V_{NMDA}-V\right)]+n_{V}(t) \end{array}$$ (2-1)

$$\begin{array}{c} \frac{dg_{*}}{dt}=\frac{\text{1}}{\tau_{*}}\;\left(\sum_{r=sp,\;inh,\;dp,ss}H\;S_{r}(V)-g_{*}\right)+n_{g_{*}}(t) \end{array}$$

$$\begin{array}{c} {}*=[L,AMPA,GABA,NMDA] \end{array}$$ (2-2)

$$\begin{array}{c} \frac{d{\left[K^{+}\right]}_{ex}}{dt}=\epsilon \left(-\gamma {\left[K^{+}\right]}_{ex}+\beta V\right)+n_{{\left[K^{+}\right]}_{ex}}(t) \end{array}$$ (2-3)

The dynamics of a neuronal membrane is given by Equation (2-1), where the membrane potential and capacitance are denoted by V, and C, respectively (the list of all acronyms and their units are provided in Tables 1–3). Constant passive leak current with a fixed conductance is denoted by $L_{c}$ and S(.) is a sigmoid transformation. A receptor time constant of a ion channel is $\tau_{*}$ (reciprocal of which is rate constants $\kappa_{*}$) and the reversal equilibrium potentials are $V_{L_{c}}$, $V_{K}$*,*$V_{NMDA},$ $V_{AMPA}$ and $V_{GABA}$. In the model, depolarisation is equipped with activity-dependent magnesium blocks, modelled as $S_{\left[Mg^{+}\right]}(V)=\frac{\text{1}}{\text{1}+\text{0}.\text{2}\;exp(-\alpha_{NMDA\;}V)}$ where the constant $\alpha_{NMDA\;}=\text{0}.\text{0}\text{6}\text{2}\;(mV^{-\text{1}})$, determines the steepness of the voltage dependence. The external inputs to membrane, is a randomly generate fluctuations with Gaussian distribution denoted by $n_{V}(t)\sim \;N(\mu_{V},\;\Sigma_{V})$.

Equation (2-2) represents the dynamics of the several conductance(s) $g_{L}$, $g_{NMDA}$, $g_{AMPA}$, and $g_{GABA}$. In equation (2-2) presynaptic firing rates (from distal populations) are denoted by$\;S_{.}(V)$, and scaled by connectivity matrix H. The connectivity matric H in equation (2-2) includes excitatory AMPA, and NMDA connection strengths ($H_{nmda/amap}$) and inhibitory GABAergic connection strengths ($H_{i}$) (see Table 3). The extrinsic input to each conductance in equation (2-2) is denoted by $n_{g_{*}}(t)\sim N(\mu_{g_{*}},\;\Sigma_{g_{*}})$ (mean and covariance are denoted by $\mu_{g_{*}}\;$and $\Sigma_{g_{*}}$, respectively).

Equation (2-3) represents extracellular potassium concentration dynamics. In the absence of any biological feedback between neuronal substrate and potassium concentration (i.e., β=0) we expect a decay with the rate of −𝛾 Hz of the ${\left[K^{+}\right]}_{ex}$ variable to its equilibrium. In equation (2-3) parameter 𝛽 is called a fast-to-slow connection, that reflect the level of the regulation between slow and fast states. The random fluctuation input to ${\left[K^{+}\right]}_{ex}$, is denoted by $n_{{\left[K^{+}\right]}_{ex}}(t)$ $\sim N(\text{0},\;\Sigma_{{\left[K^{+}\right]}_{ex}})$ (covariance is denoted by $\Sigma_{{\left[K^{+}\right]}_{ex}}$) and the time scale separation constant between slow and fast states dynamics is denoted by ϵ.

We consider laminar specific dynamics of ${\left[K^{+}\right]}_{ex}$ that regulates layer-specific neuronal dynamics [28]. The layer-specific interactions between slow and fast states allows the simulation of laminar specific DC level shifts across the layers as well as paroxysmal transitions. The infra-slow oscillations dynamics levels in electrophysiological recordings are different across the layers and provide information about the seizure dynamics that otherwise could not be gained by conventional (i.e., high passed filter) recordings.

We mathematically model ISO as a low pass filtered proxy of ${\left[K^{+}\right]}_{ex}$ trajectory. Biophysically, increases in ${\left[K^{+}\right]}_{ex}$ result from heightened synaptic activity and impaired clearance mechanisms. The temporal integration of ${\left[K^{+}\right]}_{ex}$ reflects the net depolarising or hyperpolarising drive over extended periods, consistent with changes in the infra-slow fluctuations.

The fast states of the model generate full-bandwidth data, while slow dynamics are related to infra-slow activity. The relation between empirical data and (hidden) slow and fast states at each layer of the cortex are given as follows:


$$\begin{array}{c} Supra\;granular\;layer:\left\{ \begin{array}{c} FB^{sg}\;=\;L_{\text{1}}(sg+{J_{\text{1}\text{2}}(g)+J_{\text{1}\text{3}}(ig))+e}_{FB,\text{1}}\;\\ ISO^{sg}\;=l_{\text{1}}(F\int {{\left[K^{+}\right]}_{ex})}_{sg}dt+e_{ISO,\text{1}} \end{array}\right. \end{array}$$ (3-1)

$$\begin{array}{c} Granular\;layer:\left\{ \begin{array}{c} FB^{g}\;=\;L_{\text{2}}(J_{\text{2}\text{1}\;}(sg)+{g+J_{\text{2}\text{2}}(ig))+e}_{FB,\text{2}}\;\\ ISO^{g}\;=l_{\text{2}}(F\int {{\left[K^{+}\right]}_{ex})}_{g}dt+e_{ISO,\text{2}} \end{array}\right. \end{array}$$ (3-2)

$$\begin{array}{c} Infra\;granular\;layer:\left\{ \begin{array}{c} FB^{ig}\;=\;{\;L}_{\text{3}}(J_{\text{3}\text{1}\;}(sg)+{J_{\text{3}\text{1}}\;g+ig)+e}_{FB,\text{3}}\;\\ ISO^{ig}\;=l_{\text{3}}(F\int {{\left[K^{+}\right]}_{ex})}_{ig}dt+e_{ISO,\text{3}} \end{array}\right. \end{array}$$ (3-3)

The left-hand terms $FB^{sg}$, $FB^{g}\;$and $FB^{ig}$ in equations (3-1) to (3-1) denote full-bandwidth data as the scaled (by sensor gains $L_{\text{1},\text{2},\text{3}}$) excitatory activities at the supra-granular (sg), granular (g) and infra-granular *(*ig), respectively. The activity at each layer, is given as the summation of the local and distal excitatory activities weighted by unknown $J_{ik}\;$(contribution of $j^{th}$ layer of the cortex into the activity of $i^{th}$ layer with i,j= 1,2,3). In equation (3-1), to (3−3), laminar infra-slow oscillations are denoted by $ISO^{sg}$, $ISO^{sg}$ and $ISO^{ig}$ which are modelled as leaky low pass transform functionals of layer specific extracellular potassium concentrations, scaled by parameters $l_{\text{1},\text{2},\text{3}}$. These transforms mean that the observables capture cyclical build‑up and clearance of ionic dynamics around baseline rather than an ever‑increasing integral. Random effects are $e_{ISO,i}$ and $e_{FB,i}$ (i=1,2,3) are modelled by identically independent distribution random in equations (3-1) to (3-3).

In principle, the relationship between the latent potassium dynamics in the model and actual extracellular potassium concentrations is governed by unknown calibration factors that would need to be established through direct potassium measurements. Accordingly, estimation of this variable should be interpreted as a latent proxy for potassium ionic dynamics and excitability in this animal model. While our estimation framework enables robust inference of the relative timing, laminar organisation, and seizure-associated modulation of this slow process, it does not permit direct quantification of absolute extracellular potassium concentrations.

### Estimation procedure of multiscale model from DC-coupled data

**Fig 3:**
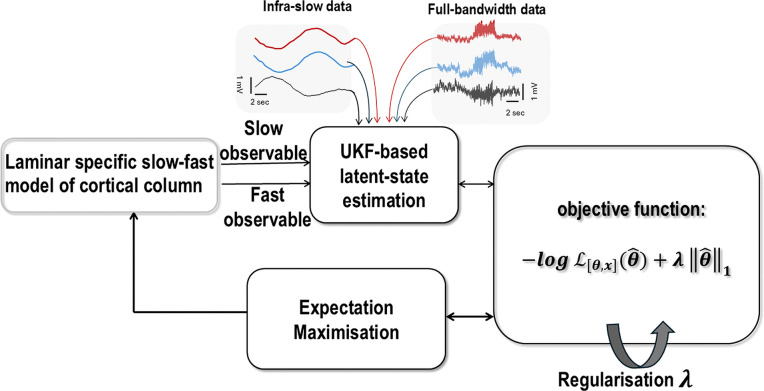
Parameter estimation framework for slow-fast model using full-bandwidth data. For each candidate set of the model parameters $\hat{\theta }$, hidden states are inferred from full-bandwidth and infra-slow oscillations data using UKF. The estimated states are then used to compute the negative log likelihood (of the observed data under the model) ${-log\;L}_{\left[\theta ,x\right]}(\hat{\theta })$, regularised by $L_{\text{1}}$ norm of the parameters through a factor λ. The regularised negative log likelihood function is optimised using expectation-maximisation algorithm for estimating the model parameters.

We developed a regularised maximum-likelihood procedure to estimate the model parameters from the DC-coupled animal recordings, as detailed in Fig 3. For each candidate set of model parameters, we utilise an unscented Kalman filter (UKF) to estimate hidden states of the model from infra-slow oscillations and full-bandwidth data. The UKF is efficient in handling distribution of latent states up to the third order of the Taylor series expansion for any nonlinearity [50] and well established for real time monitoring and control [51]. Deterministic sampling known as unscented transform is used as part of the UKF to accurately capture the statistical properties of nonlinear transformations. This enables efficient estimation of hidden states distribution, facilitating calculation of the model likelihood [29,33].

We introduce $L_{\text{1}}$ regularisation (on parameters norm) into the negative log-likelihood function to reduce the possibility of overfitting and improve generalisation. The regularisation parameter is selected based on Akaike information criterion (AIC) scores through greedy search where the first minimum amongst AIC scores is selected as the regularisation parameter. We consider mean field decomposition between sensor noise parameters and biological parameters due to their statistical independence.

Parameter estimation based on the likelihood procedure is performed using an expectation-maximisation (E-step and M-step) algorithm. In the E-step, the UKF is used to estimate the states of the model from the observables (i.e., full-bandwidth data as the observation of fast states and infra-slow oscillations as the observation of slow states). After estimating the distribution of the hidden states, we construct the likelihood function and perform the M-step which involves estimation of model parameters and estimation measurement noise covariance.

In the M-step, we first assume the measurement noise distribution and optimise the likelihood of the model with respect to model parameters. The resulting estimates are then used to update the sensor noise parameters, and this process is repeated until the convergence criteria are met. Next, the overall estimate of constant parameters is fed back into the E-step, and the iterations continue until no further improvements in the likelihood function. The EM is then carried out by introducing different regularisation and their efficacy is assessed through model comparison based on AIC.

We perform Monte Carlo experiments, e.g., [52], by re-estimating the model for different ${\left[K^{+}\right]}_{ex}$ in the range [3.5−6] Mm. The mean and standard deviation of estimated parameters are then considered as the inference results. Using these estimations, we evaluate the consistency of tracking the slow potassium dynamics over the course unseen data segments.

## Results

### Model simulation

**Fig 4:**
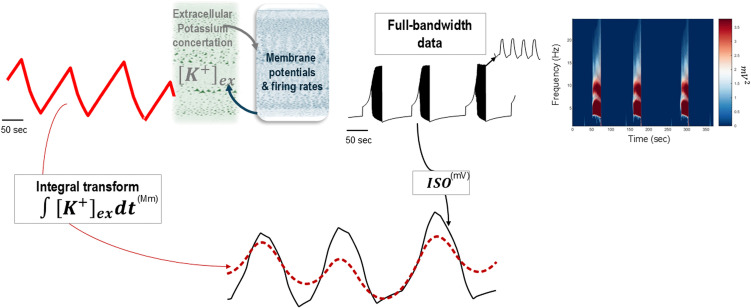
Simulation of the slow-fast cortical column model reproducing seizure-like dynamics. The centre panel shows the slow-fast cortical column model in which reciprocal feedback between ${\left[K^{+}\right]}_{ex}$ and neuronal activity drives transitions into and out of seizures. The left panel illustrates simulated ${\left[K^{+}\right]}_{ex}$ dynamics associated with the simulated full-bandwidth data reflecting 5-10 Hz SWDs characteristic of absence seizures in the right panel. The AC-coupled (high passed filter) data and time-frequency plots highlight the emergence of SWDs during seizure episodes. The bottom panel illustrates the relationship between the (leaky) integral transform of ${\left[K^{+}\right]}_{ex}$ as an ISO proxy. The model reproduces the gradual onset of seizures driven by slow ionic changes, consistent with the observed infra-slow activity in the WAG-Rij animal data.

We first simulate the slow-fast model to examine whether slow extracellular potassium dynamics could induce seizure-like transitions. The simulation is shown in Fig 4, where build-up in ${\left[K^{+}\right]}_{ex}$ triggered transitions into spike-and-wave discharges (SWDs) in the model’s output. This closely resemble the electrographic phenotype observed in the WAG-Rij rat model. The simulated SWDs exhibited 5−10 Hz oscillations which were preceded by infra-slow changes. The model produced a coupling between the integral transformed ${\left[K^{+}\right]}_{ex}$ and ISO, demonstrating that slow ionic dynamics are sufficient to generate seizure-like transitions endogenously.

### Parameter estimation from full-bandwidth data

**Fig 5:**
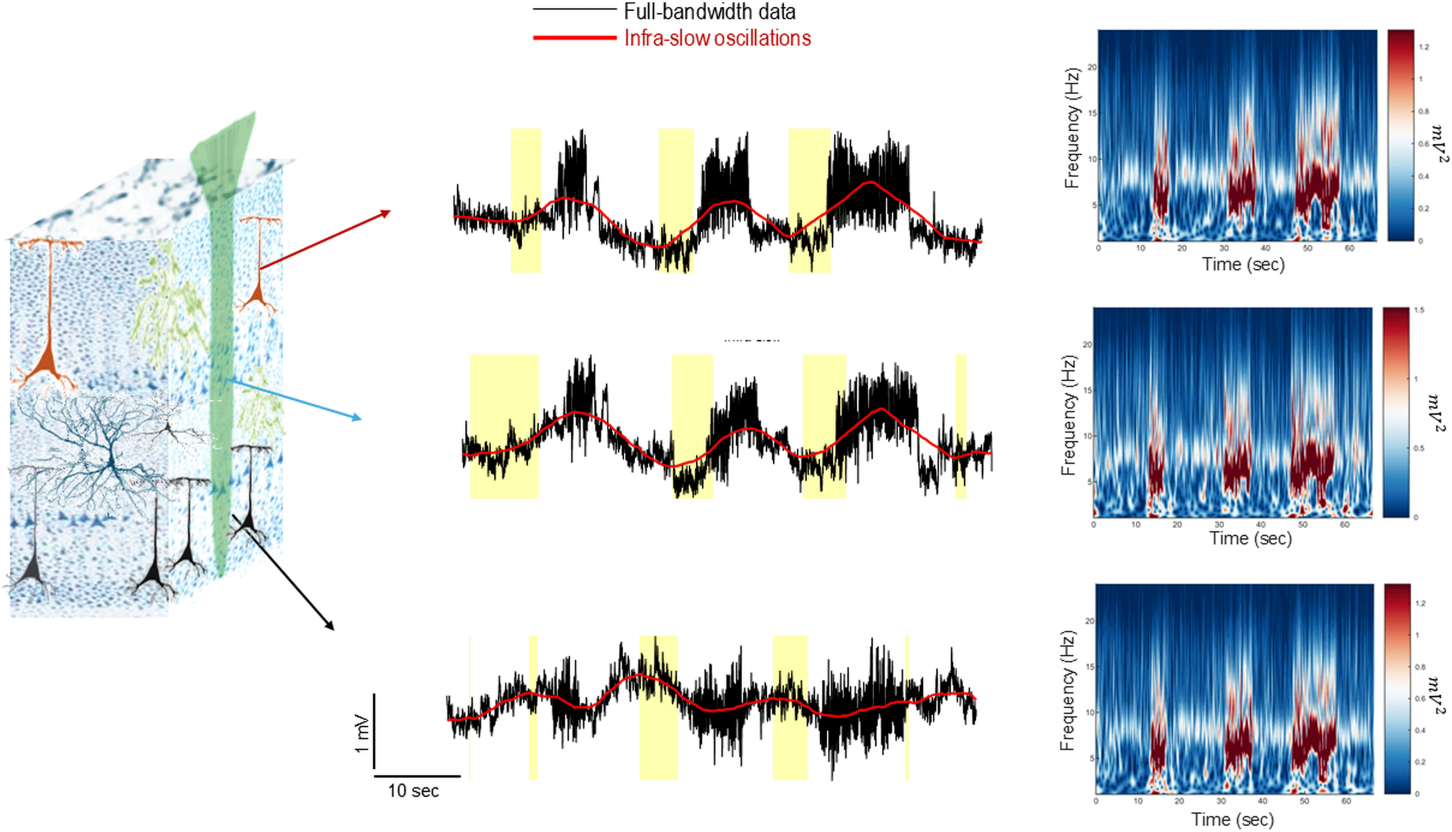
Laminar recordings and ISO preceding seizures in the WAG-Rij rat model. DC-coupled recordings from the somatosensory cortex of a WAG-Rij rat, obtained using a 16-channel linear array of graphene solution-gated field-effect transistors. The recordings include layer-specific full-bandwidth local field potentials (black lines) including ISOs (red lines). In the middle panel, laminar traces show the spatiotemporal structure of seizure-related activity. The yellow-shaded regions indicate the onset of infra-slow oscillatory phases that precede seizure initiation, reflecting slow ionic shifts that build up prior to the emergence of epileptiform activities. The data show layer-dependent DC shift before seizure onset. The right panel shows time frequency plots of the data which highlights seizure-associated SWDs in the 5-10 Hz range (the sampling frequency of the recorded data is 9.6 kHz).

We next apply the regularised likelihood procedure (Fig 3) to estimate model parameters from DC-coupled laminar recordings and ISOs as shown in Fig 5. Parameters are estimated using the expectation-maximisation algorithm with the UKF for latent-states estimation [29].

Specifically, we estimate all parameters governing the slow ${\left[K^{+}\right]}_{ex}$ dynamics (i.e., layer-specific time constants, slow-to-fast connectivity, extrinsic inputs and time scale separation), the inputs to the fast sub-system, the contribution of distal layer activity to each cortical layer’s readout (interlaminar mixing weights), and sensor-noise parameters. The initial estimate for the time-scale separation parameter is the average inter-seizure intervals. All remaining parameters not directly related to ${\left[K^{+}\right]}_{ex}$ dynamics are fixed at biologically plausible values to reduce inference complexity. The regularisation weight λ is systematically varied over [0−10] and set equal to 4 based on the first minimum amongst AIC scores.

To assess robustness, we perform 100 Monte Carlo simulations by varying the initialisation of ${\left[K^{+}\right]}_{ex}$ within [3.5−6] mM. We provide the summary statistics (mean and standard deviation) in Table 3, which indicate tight distributions in the estimated parameters, particularly those related to extracellular potassium time constants and layer-specific connectivity. Note that the inferred slow-state dynamics should be interpreted as a latent proxy for potassium ionic dynamics and excitability. In the present framework, they represent ISO-constrained latent mesoscale states rather than direct ion-selective measurements. In effect, their principal value lies in their relative temporal evolution, laminar organisation, and coupling to seizure transitions, rather than in precise absolute concentration estimates.

**Fig 6:**
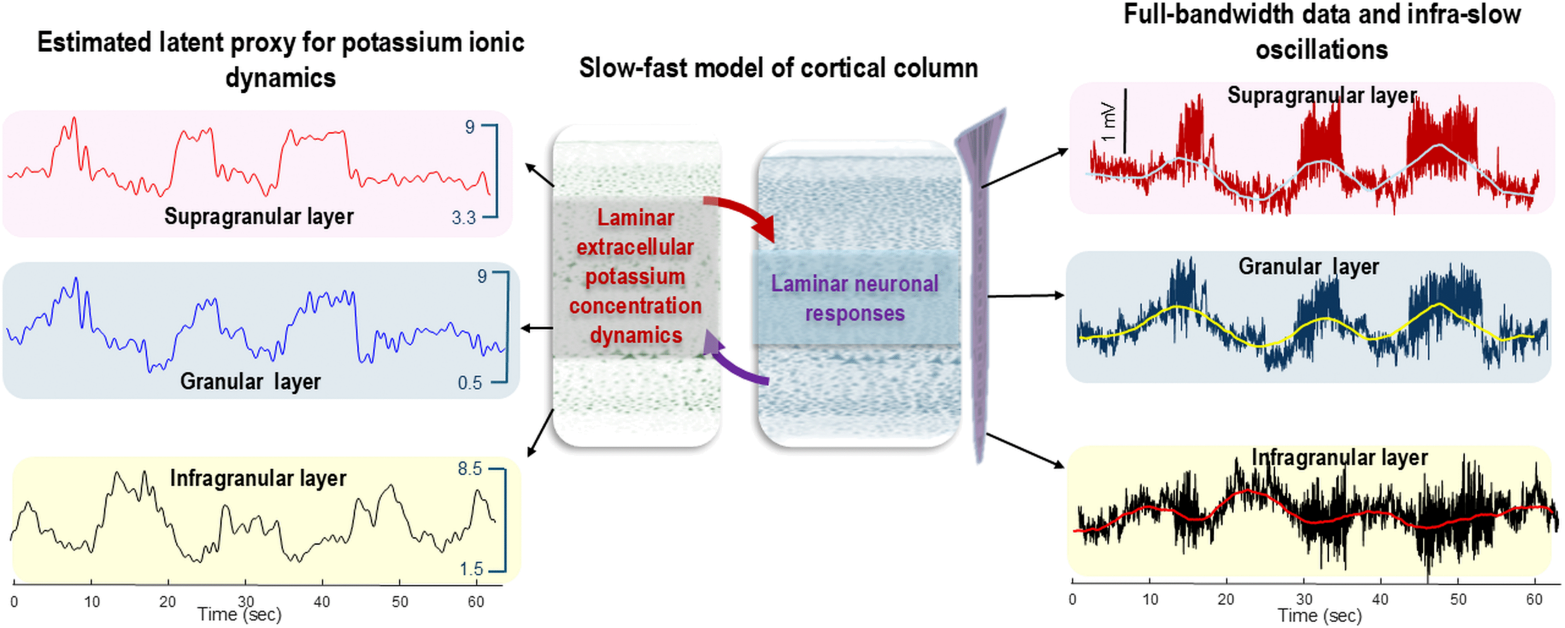
Layer-specific estimation of latent proxy for potassium dynamics form DC-coupled data. Right panel shows laminar DC-coupled data and the corresponding infra-slow oscillations. Estimated latent proxy for extracellular potassium concentrations at each layer are shown in the left panel using the inference procedure. The results reveal distinct, layer-specific ionic dynamics, with approximately reciprocal patterns observed between superficial and deep layers. Quantitatively, the mean (± variability) of ${\left[K^{+}\right]}_{ex}$ in the superficial layer is 6.1  (±2.7), in the granular layer 4.7  (±4) and in the deep layer 5 (±3.2). These support the role of laminar ${\left[K^{+}\right]}_{ex}$ in driving seizure transitions. Note that the inferred latent potassium dynamics are reported as dimensionless, because the calibration factors relating the latent slow states to actual extracellular potassium concentrations are unknown.

Inferred latent ${\left[K^{+}\right]}_{ex}$ trajectories are shown in Fig 6. These estimates show a reciprocal pattern of ${\left[K^{+}\right]}_{ex}$ between superficial and deep layers, consistent with prior physiological studies [28]. The slow evolution of latent potassium levels correlates with transitions into and out of seizure states, supporting the hypothesis that paroxysmal dynamics arise from intrinsic changes in extracellular potassium concentrations.

### Reproducibility and consistency of inference over unseen data

**Fig 7:**
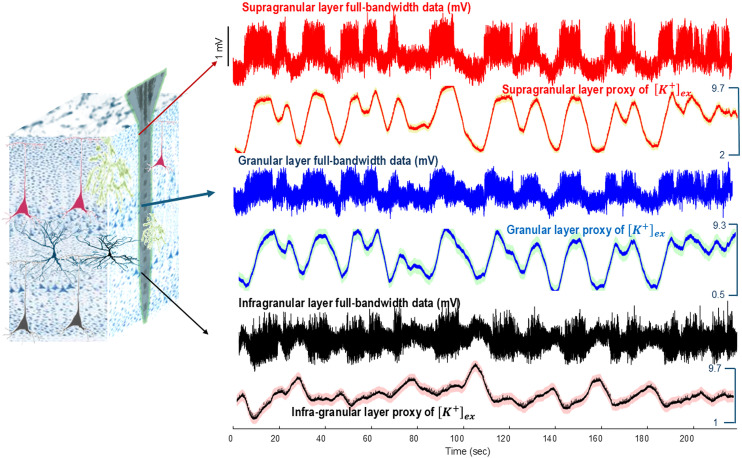
Consistency and reproducibility of inferred latent proxy of potassium dynamics across unseen data. Estimated expected values of ${\left[K^{+}\right]}_{ex}$ from a 220 sec, DC-coupled, full-bandwidth recording not used for parameter estimation, aggregated over 100 Monte Carlo runs. Layer-specific dynamics remain consistent with the training set. The plots show mean trajectories of ${\left[K^{+}\right]}_{ex}$ across superficial granular, and deep cortical layers, aggregated over 100 Monte Carlo simulations demonstrating generalisability. Mean(± variability) of ${\left[K^{+}\right]}_{ex}$ at: Superficial layer: 5.8 (±3.7), Monte Carlo variation = 1.5%. Granular layer: 4.8 (±4.2), Monte Carlo variation = 2.0%. Deep layer: 5.3 (±4.1), Monte Carlo variation = 3.4%. The reciprocal relationship between superficial and deep layer dynamics is preserved, underscoring the robustness of the estimation procedure. These findings further validate that the model can reliably track latent ionic dynamics from full-bandwidth DC-coupled data and supports its utility for real-time seizure monitoring. Note that the inferred latent potassium dynamics are reported as dimensionless, because the calibration factors relating the latent slow states to actual extracellular potassium concentrations are unknown.

To assess the generalisability, we used a 220 second held-out data segment (not used during estimation) to reconstruct trajectories of extracellular potassium concentrations from the infrared models. Fig 7 overlays the estimated ${\left[K^{+}\right]}_{ex}$ trajectories from the 100 Monte Carlo simulations. The inferred trajectories are consistent with those obtained on the training data, demonstrating reproducible dynamics and supporting the construct validity of the model. Across layers, the model achieved low variance and minimal inter-simulation deviation. This consistency across both training and unseen data confirms that ISOs encodes reliable information about extracellular potassium concentration dynamics, and that the model can generalise beyond specific seizure instances.

## Discussion

We present a data-driven multiscale modelling framework capable of inferring hidden ionic dynamics underlying paroxysmal transitions in an animal model of absence epilepsy. Our innovation lies in leveraging variability in infra-slow oscillations, as an indirect proxy for the dynamics of transformed latent slow ionic/excitability states, alongside full-bandwidth data as the readout of fast neuronal activities. This approach increases available data points and information available for tracking hidden biological states and improves the identifiability, enabling accurate tracking of physiological processes that drive seizure transition. By modelling ISOs as the low-passed transformed proxy of latent extracellular potassium concentrations, our framework captures bifurcation properties of seizure dynamics. In fact, the slow-fast model can generate phase transitions with or without changes in infra-slow oscillations that are induced by sudden and smooth transitions in slow states, respectively, e.g., [28,53]. Linking infra-slow oscillations to the transformed latent slow excitability states not only provide a mechanistic interpretation of slow cortical fluctuations but also offers a biophysically grounded constraint for parameter inference.

Our data-driven modelling enables decomposition of seizure transitions into interpretable phases of build-up, onset, and resolution. Unlike AC-coupled data, which often removes pre-ictal shifts, DC recordings reveal gradual changes that are essential for reconstructing the full trajectory of latent causes of the paroxysmal events. The separation of fast and slow components, constrained by ISOs, is analogous to dual Kalman filtering for nonlinear parameter estimation [54], but with a more physiologically grounded evolution pattern. Our results demonstrate that inferred proxy of ${\left[K^{+}\right]}_{ex}$ dynamics vary across cortical layers and evolve on timescales compatible with ISOs. These slow shifts precede seizure onsets and exhibit reciprocal patterns between superficial and deep layers, consistent with previous experimental findings [28,30,31].

However, while our model assumes that ${\left[K^{+}\right]}_{ex}$ dynamics drive ISOs and contribute to seizure initiation, we cannot rule out the possibility that ISO is correlative rather than causal with slow ionic dynamics. In vivo DC-coupled electrophysiology recordings combined with potassium-selective microelectrodes measurements will be required to test this empirically. However, measuring extracellular potassium concentration dynamics in the awake brain with ion-selective probes is technically challenging and, to the best of our knowledge, has not been reported; such measurements are typically conducted in acute brain slices**.** Accordingly, our contribution should be understood primarily as a novel hypothesis‑driven identification of slow-fast dynamical systems rather than definitive empirical validation of potassium-driven causality.

The mesoscale model can be seen as an abstraction of more detailed computational models [17,55–57]. For instance, the extended Hodgkin–Huxley model developed by Wei, Ullah [56] unifies neuronal spikes, seizures, and spreading depression (see Supplementary information). While our model simplifies these processes by abstracting energetic constraints, it retains the essential link between the extracellular potassium concentration and seizure onset, enabling efficient inference and potential real-time application.

The abstraction of the mesoscale slow–fast model comes with limitations. It does not capture all biophysical mechanisms (e.g., glial–neuronal volume regulation or oxygen-dependent ATPase dynamics), nor does it include multimodal data such as calcium imaging or hemodynamic signals for the inference. Future work could incorporate these modalities, and use advanced transformation mappings for nonlinear filtering of dynamical systems [51,58] to compensate for model inadequacy.

Despite these limitations, our results suggest that monitoring ISOs as proxy measurements for seizure susceptibility windows could support the development of targeted intervention strategies. From a control-theoretic perspective, relative changes in ${\left[K^{+}\right]}_{ex}$, rather than their absolute measurements, may be sufficient for seizure prediction and treatment optimisation [51,57]. Practical control systems tuning (e.g., adjusting observer gains) could compensate for uncertainties in absolute ${\left[K^{+}\right]}_{ex}$ [51]. In addition, monitoring ${\left[K^{+}\right]}_{ex}$ within the neuronal system may suffice for online monitoring seizures, as empirical observation suggested dynamics of ${\left[K^{+}\right]}_{ex}$ and glial activities are consistently changed during the paroxysmal transitions [17,22,23,59–61].

Finally, the WAG-Rij rat model is pharmacologically responsive to human absence epilepsy treatments, making it a valuable translational platform. The consistency of inferred dynamics across unseen data suggests that our generative model effectively captures mechanisms of seizure dynamics. As such, this approach may be extended to human intracranial recordings to support real-time seizure tracking in clinical settings.

## Supporting information

S1 Appendix A detailed biophysical model of paroxysmal transitions. This supporting information file contains the supplementary description of the extended Hodgkin-Huxley model simulation and includes Fig S1, showing membrane dynamics, extracellular potassium concentration dynamics, and the relationship between ISO and the transformed extracellular potassium signal.
(DOCX)
