# Supplementary material for: Data driven multiscale modelling of paroxysmal brain transitions using DC-coupled electrophysiological data
Source: PLoS One. 2026 Jul 17;21(7):e0353399. doi: 10.1371/journal.pone.0353399 (PMC13379037; doi:10.1371/journal.pone.0353399)
Supplement: S1 Appendix — This supporting information file contains the supplementary description of the extended Hodgkin-Huxley model simulation and includes Fig S1, showing membrane dynamics, extracellular potassium concentration dynamics, and the relationship between ISO and the transformed extracellular potassium signal. (DOCX) [file pone.0353399.s001.docx]

## Supplementary information: a detailed biophysical model of paroxysmal transitions

Wei et al. (2014) extended the Hodgkin–Huxley model to capture a broad range of neuronal dynamics, including action potentials, seizures, spreading depression, and hypoxia-induced depolarisation. Their model integrates ion homeostasis, energy metabolism, and neuronal excitability to explore the generation, maintenance, and potential reversal of pathological brain states. Notably, they demonstrated that seizures and spreading depression, though traditionally viewed as distinct, arise from a shared biophysical basis.

The model incorporates mass and charge conservation and explicitly links the energetic cost of ion transports $\left[ {Na}^{+} \right]$ /$\left[ K^{+} \right]_{ex}$ activity to oxygen availability. This reveals seizures and spreading depression as points along a continuum of membrane excitability regulated by potassium levels and metabolic constraints.

The model captures dynamic changes in intra- and extracellular sodium, potassium, and chloride concentrations, influenced by voltage-gated ion channels, ion pumps, leak currents, glial buffering, and diffusion to both neighbouring cells and the vasculature. The $\left[ {Na}^{+} \right]$ /$\left[ K^{+} \right]_{ex}$ pumps and glial potassium uptake are modelled as oxygen-dependent, allowing simulation of both physiological and pathological states under normoxic and hypoxic conditions. Additionally, the model incorporates osmotic volume regulation, where the ion redistribution drives cellular swelling and extracellular space shrinkage, further modulating excitability. This accounts for dynamic intracellular-to-extracellular volume ratios based on osmotic pressure gradients, consistent with experimentally observed volume changes during spreading depression.

A simulation of the model is given in Fig S1 where the system exhibits resting states, but the changes in extracellular potassium concentrations (approximately $8-12 mM)$ drives seizure-like oscillations. More crucially, we re-confirm the link between $ISO$ and integral transformed of potassium level with this model as shown in Figure 1S.


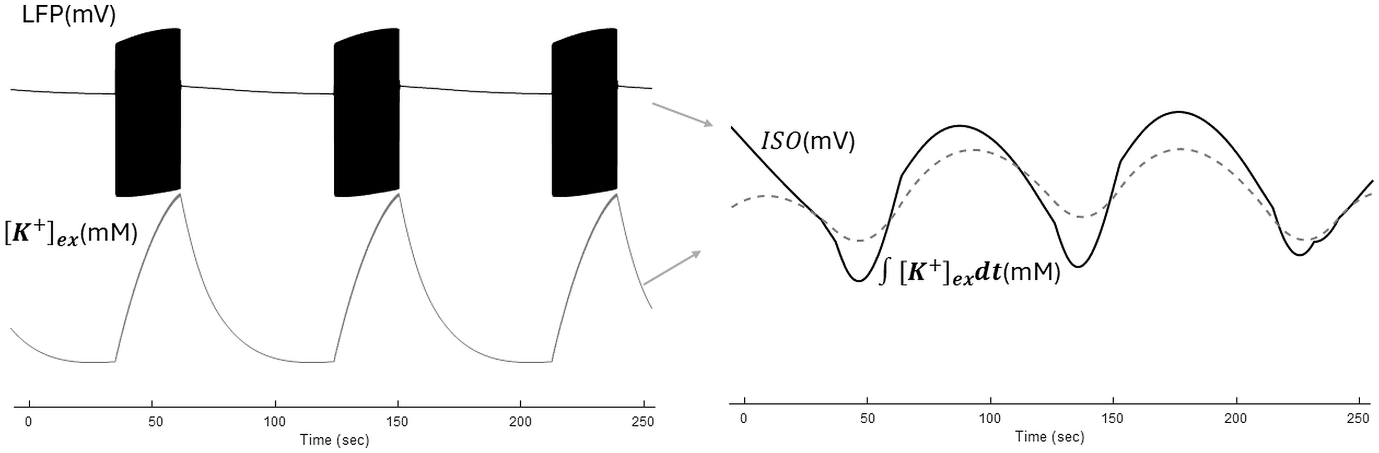


*Fig S1:* ***A simulation of an extended Hodgkin–Huxley model (Wei et al., 2014)****. The left panel shows the membrane neuronal dynamics (into and out of seizures) and the extracellular potassium concentrations. The right panel illustrate the consistency between ISO (solid line) and the integral transformed of the extracellular potassium concentration (dashed line). To maintain consistency with the pathophysiology of seizures in the animal model, the dynamics of other ion levels are considered constant in the detailed neuron model. The code for the model was provided in https://tinyurl.com/433jh23.*

WEI, Y., ULLAH, G. & SCHIFF, S. J. 2014. Unification of neuronal spikes, seizures, and spreading depression. *Journal of Neuroscience,* 34**,** 11733-11743.
